# Rethinking advanced motherhood: a new ethical narrative

**DOI:** 10.1007/s11019-023-10172-w

**Published:** 2023-09-03

**Authors:** Eva De Clercq, Andrea Martani, Nicolas Vulliemoz, Bernice S. Elger, Tenzin Wangmo

**Affiliations:** 1 Institute for Biomedical Ethics, University of Basel, Bernoullistrasse 28, 4056 Basel, Switzerland; 2 CPMA Lausanne, Rue de La Vigie 5, 1003 Lausanne, Switzerland; 3 Center for legal medicine, University of Geneva, Geneva, Switzerland

**Keywords:** Delayed childbearing, Older motherhood, Reproductive ethics, Infertility, Feminism, Vulnerability

## Abstract

The aim of the study is to rethink the ethics of advanced motherhood. In the literature, delayed childbearing is usually discussed in the context of reproductive justice, and in relationship to ethical issues associated with the use and risk of assisted reproductive technologies. We aim to go beyond these more “traditional” ways in which reproductive ethics is framed by revisiting ethics itself through the lens of the figure of the so-called “older” mother. For this purpose, we start by exploring some of the deep seated socio-cultural discourses in the context of procreation: ageism, ableism and the widespread bias towards geneticism and pronatalism. Afterwards, we provide a critical overview of the key arguments against or in support of advanced motherhood. We then briefly discuss how entrenchment by both sides has produced an impasse in the debate on the ethics of advanced motherhood and proceed by arguing that it is fundamental to bring about a change in this narrative. For this purpose, we will revisit the feminist usage of the concept of vulnerability which will allow us both to criticize culturally prescribed norms about motherhood and to address the painful reality of age-related fertility decline. In the last section, we argue that instead of defining “older” motherhood as an ethical problem, we should problematize the fact that female reproductive ageing is an understudied and ill-sourced topic. We believe that allocating resources to research to better understand female reproductive ageing is not only ethically permissible, but might even be ethically desirable.

## Introduction: delayed childbearing and advanced motherhood, a worrying trend?

Childlessness is becoming a widespread phenomenon in middle and high-income countries (Sobotka & Beaujouan 2017, 2019). It is often discussed alongside delayed childbearing since advanced maternal age is one of the leading explanations of infertility and reduced reproductive potential. Although childbearing at older age was a common social practice until the 1960s (Desjardins et al. 1994, Friese et al. 2008), the new feature is that the mean age of women at *first* birth has increased progressively over the last few decades, and that reproduction is often obtained through medical means (Baldwin 2019).

A high percentage of infertile individuals report physical, psychological and sociological problems that might severely compromise their quality of life (Hazlina et al. 2022, Kiani et al. 2021, Luk et al. 2015). Age-related infertility and delayed childbearing also lead to broader demographic and economic concerns for governments as lower birth rates imperil economic growth and the sustainability of welfare systems (Kramer 2014). These negative outcomes explain why delayed childbearing is increasingly perceived as not just a private, but also a *public* health issue (Balasch & Gratacós 2012; Lemoine & Ravitsky 2015). As a result, tackling the problem of age-related infertility has become a pressing and substantial objective in many countries. The measures to address the problem may be technological (e.g. improvements in assisted reproductive technology (ART)), but also social and political in nature (e.g. fertility awareness campaigns, financial incentives for families with children, improved child-care services etc.).

In this contribution, we aim to rethink the ethics of advanced motherhood by arguing that we need a new ethical narrative that problematizes the fact that female reproductive ageing is an understudied topic. In the literature, delayed childbearing is usually discussed in the context of reproductive justice, and in relationship to the ethical issues and risks associated with the use of ART (e.g. inequitable access, lack of regulatory bodies, safety, pre-genetic screening). We aim to go *beyond* these more “traditional” ways in which reproductive ethics is framed: instead of focusing on ethical issues related to delayed childbearing (e.g. harm to the child) and the means of conception (i.e. ART), we aim to revisit (reproductive) ethics itself through the lens of the figure of the so-called “geriatric” mother. Thus, rather than considering advanced motherhood as a “special” case, we aim to take on the subject position of the “older” mom. For this purpose, we will start by exploring some of the deep seated socio-cultural discourses in the context of procreation: ageism, ableism and the widespread bias towards geneticism and pronatalism. Afterwards, we provide a critical overview of the key arguments against or in support of advanced motherhood. We then briefly discuss how entrenchment by both sides has produced an impasse in the debate on advanced motherhood and proceed by arguing that it is fundamental to bring about a change in the narrative on the ethics of advanced motherhood. For this purpose, we will revisit the feminist usage of the concept of vulnerability which will allow us both to criticize culturally prescribed norms about motherhood and to address the painful reality of age-related fertility decline. In the last section, we argue that instead of defining “older” motherhood as an ethical problem, we should problematize the fact that female reproductive ageing is an understudied and ill-sourced topic. We claim that allocating resources to research to better understand female reproductive ageing is not only ethically permissible, but might even be ethically desirable.

Before we proceed, we want to make three remarks. First, the decision to focus on women does not imply that the topics of advanced *paternal* age and *male* infertility are unimportant. Still, we should not forget that the cultural discourse on advanced parenthood is profoundly “gendered”. This is due to women’s specific role in reproduction, on the one hand, and the dominant cultural imperative of motherhood, on the other hand. Our focus on motherhood stems also from another reason: for males, fertility does not decrease with the same degree as for females. Second, throughout the paper we will focus primarily on persons who become pregnant at an age which, from a medical perspective, is considered “old” due to increased health risks for both mother and child. Hence, issues related to adoption will be set aside. In no way does this imply that we want to favor genetic and gestational motherhood at advanced age over adoption at later age. Third, by no means do we want to minimize the impact of age on infertility and health risks for both mother and child. The focus rather lies on the following question: given these undeniable medical risks, is it ethically problematic to become a mother at an advanced age?

## Deep-seated socio-cultural discourses on motherhood

### The pronatalist imperative and genetic fetishism

In contemporary society, having children is generally considered to be the default position: it is expected that most members of society will (and want to) have children, and that if they don’t, they need to provide a justification (Overall 2013). This is particularly the case for women as in many cultures motherhood is still considered as central to womanhood, to the extent that mothering is not a choice, but rather a social imperative (Parry 2005; Petropanagos 2017; Warnes 2019). Still, as Overall convincingly argues in *Why have children*? (2013), maybe the burden of proof should be inverted: from an ethical point of view the choice to procreate is far from “natural”. On the contrary, it is a decision that calls for a rigorous ethical analysis, because it involves the bringing into existence of a new vulnerable being and greatly impacts the wellbeing of parents and other family members (Overall 2013).

Pronatalism, i.e. the social bias that promotes childbearing, permeates not only state policies, but everyday cultural discourses as well (Bell 2019; Scala & Orsini 2022; Warnes 2019). Women who have not (yet) reached motherhood are regularly confronted with the invasive question: «When are you going to have kids?» (Wells & Heinsch 2020). The hidden message in the question is that women are either deliberately delaying childbearing and thus challenging their biological clock with all the multiple risks involved or deliberately putting off something which is considered to be socially desirable. Studies have shown how stigmatizing and stressful this experience can be, as it makes women feel selfish and incomplete (Bartholomaeus & Riggs 2017; Gentile 2013) and might place an important strain upon relationships (Locke et al. 2013). Motherhood is still assumed to be one of the main ways to find meaning, satisfaction and happiness in adulthood (Gotlib 2016). Hence, women who are childless are perceived as missing out on something fundamental. This view is somehow reinforced by language, insofar women without children continue to be labelled in reference to a child, and are considered “less” without them (Gotlib 2016; Gouni et al. 2022).

On top of being pronatalist, contemporary western society also privileges genetic ties in relation to procreation (Petropanagos 2017). Geneticism, i.e. the social bias towards genetic genealogical relationships, is heavily reflected in the social language surrounding maternity. Within society, infertility, in fact, is often equated with the incapacity of having a child *of* one’s own rather than the incapacity to have a child *on* one’s own (Brakman & Scholz 2006). There is a strong social tendency to consider genetic relationships as the most desirable and natural: they are deemed constitutive of both individual and familial identity. The social bias towards genetic relationships is also apparent in the assumption that donor-conceived children will face psychological problems as they miss crucial information to complete their self-identity (Leighton 2012). Still, existing empirical research challenges this assumption: the knowledge of one’s genetic origins does not seem to be relevant for the psychological development of adult donor offspring (Pennings 2021).

Geneticism undoubtedly shapes people’s reproductive choices and options (Bell 2019; McLeod 2017; Segers et al. 2019). In the case of infertility, many aspiring (older) mothers will first try to use their own genetic material (homologous IVF), only then opt for oocyte donation (heterologous IVF) and consider adoption as a viable option only when all medical treatments have failed (Brakman & Scholz 2006). Although gestational mothers cannot fulfill the ideal of genetic motherhood, they can still *appear* to be the natural, biological mother and thus avoid social stigmatization (Friese et al. 2008). The pressure to achieve genetic or at least biological motherhood might compel individuals to undergo mentally, physically, and financially demanding medical treatments (some with modest success rates) that «they might not otherwise have chosen» (Petropanagos 2017, p. 135). Hence, rather than enhancing women’s reproductive autonomy, advanced motherhood through ART might actually reduce women’s freedom and choice (Sperling 2012) as it might reinforce the social expectation (and the internalization of it) that women need to become mothers at all costs, (Brakman and Scholz 2006, Cutas & Smajdor 2015, Landau 2004). Also, some ART (e.g. use of donor eggs, surrogacy) might risk to exploit socially and economically marginalized women (Nahman 2018). The availability of and access to ART do thus not necessarily promote women’ autonomy as a common liberal view of reproductive autonomy might make us believe (Lee 2022).

### At the intersection of ageism and ableism

Ageism is generally defined as the stereotyping, prejudice, and social oppression towards people based on their age (Officer et al. 2018). Most of the current literature on ageism focuses on older adults aged 60 or older. Still, ageism can affect any age group, thus also middle-aged adults (de la Fuente-Núñez et al. 2021). Feminist writer Margaret Gullette (1997) describes “middle-ageism” as a socially constructed disease of the twentieth century: middle age is portrayed as a phase of decline in which beauty and health fade away (Lahad & Hadsen 2016). Discourses of ageing in terms of decline have important gendered connotations (Sandberg 2013). Women, and “older” mothers are particularly targeted in these decline narratives of middle-ageism due to cultural expectations of the “normal” mother as being young, abled and self-sufficient (Frederick 2017; Scala & Orsini 2022). Although it may seem as if the concept of successful ageing challenges ageism by emphasizing the autonomy, activity and productivity of ageing persons; it actually encourages women to hide all visible signs of ageing and to become age-less (Pilcher 2020, Sandberg 2013). This may explain why 40 plus mothers tend to dissociate themselves from the label of “being old” and do anything to appear and feel young (Lahad & Hadsen 2016).

Ageism often intersects with ableism that devalues and discriminates against people with physical, intellectual, or psychiatric disabilities (Overall 2006; Rabheru 2021). Within the context of advanced maternal age, most of the literature focuses on the risk of disability of the child or the mother (Cardin 2020; Scala & Orsini 2022). By having children at the “right time”, women can mitigate this risk. The implicit message is that «bringing disability into the world» (…) «is unwelcome, irresponsible, or tragic» (Scala & Orsini 2022, p.3).

## Advanced motherhood at the intersection of gender and age

Although women as a group are generally encouraged to pursue (genetic) motherhood, and the choice to procreate is not perceived as something in need of explanation, there are important exceptions to this (Wells & Heinsch 2020). The reproductive rights of some groups are frequently contested, like those of people with disabilities, persons who identify as LGBTQI, individuals who are single or do not have the “appropriate” age, but are considered either too young or too old to parent. As stated above, throughout this manuscript we will focus mostly on the last category, and in particular on “older” women. That does not mean to deny that some of these groups might overlap (e.g. an older woman with a disability, or who identifies as lesbian) or to claim that the challenges of these other groups are less important.

### Advanced motherhood, a risky enterprise

Much of the literature on advanced motherhood urges for caution and even rejection of childbearing at a later age (Bewley et al. 2005, Balasch & Gratacós 2012, Caplan 2010, Epstein & Zosmer 2015, Zweifel et al. 2012, 2020). The implicit message is that women/individuals of “a certain age” should not seek motherhood, and that society should not cover the cost of expensive (often unsuccessful) ART treatments in the case of age-related infertility. Instead of referring to sheer chronological age, critics of late motherhood ground their arguments usually in the child’s best interest principle. In particular, they focus on the mother’s (in)capacity to contribute to the “flourishing” of the child which is composed of different types of well-being: physical, emotional, psychological and social.

Many of the debates about late childbearing focus on medical and health-related concerns for offspring and birthing persons (Berntsen et al. 2019; Pettersson et al. 2020). Commonly reported perinatal risks of advanced motherhood are pregnancy loss, stillbirth, pre-term birth, caesarean delivery, risk of stroke, gestational hypertension, post-natal depression and death (Cavazos-Rehg et al. 2015, Correa-de-Araujo et al. 2021, Lean et al. 2017, Saccone et al. 2022, Strelow et al. 2018). Possible adverse postnatal outcomes in children can be both mental and physical in nature (Carslake et al. 2017, D’Onofrio et al. 2014, Lampi et al. 2013).

Another widely found concern is that children raised by older parents have a higher likelihood of having to take on a caregiving role for their older parents early in life, and might even face parental death before they reach the age of maturity (Zweifel et al. 2012, 2020). Both situations can take a heavy toll on the psychological, emotional and social wellbeing of children due to their negative impact on education, career plans and interpersonal relationships (Zweifel 2020). These negative outcomes might be worsened by the lack of siblings and the deprivation of grandparents at a young(er) age, two circumstances which are likely to occur to children born to older parents.

A third worry concerns directly the mother’s perceived capacity to be a genuinely good mother. It is often presumed (Shaw & Giles 2009; Zweifel et al. 2012) that older mothers have a reduced capacity to contribute to their child’s flourishing as their diminished physical and cognitive abilities make it challenging to cope with the stress associated with child rearing and to practice, what is called, “intensive mothering”, i.e. child-centered, expert-guided, emotionally absorbing, labor-intensive, and financially expensive (Hays 1996).

A final concern regards ART donor-conceived children. Although some health risks might be mitigated in the case of egg donation, as stated above, the presumption is that these children will face important identity issues due to the fact that they are not genetically related to the birth mother (Velleman 2005).

### In defense of advanced motherhood

Those who consider advanced motherhood to be ethically viable, contend that these seemingly objective and medical findings with regard to the risks of advanced maternal age are skewed by culturally dominant discourses (i.e. pronatalism, geneticism, ageism and ableism) that dominate the female body and have put forward various arguments to rebut the considerations above (Ekberg 2014, Hallgrimsdottir and Benner 2014). First of all, they question whether advanced motherhood is really the outcome of a personal, life-style choice (Cutas & Smajdor 2015). Research increasingly shows that rather than *actively* delaying childbearing in order to prioritize their education and career, most women do not deliberately choose to become mothers a later age (Cook et al. 2012). Advanced motherhood is rather the outcome of life circumstances that fall *outside* of a woman’s control, like finding a suitable partner or a stable job (Baldwin 2019). Therefore, it might be problematic to portray older mothers as *deliberate* risk-takers.

Another strategy used to counter the concerns of critics, is to put the reported medical risks for offspring into perspective (Cardin 2020, Smajdor 2011). Existing research provides contradictory evidence with regard to whether the increased risk for major morbidities is statistically relevant. The financial and relational stability of older mothers together with the surrounding medical context might compensate for poorer birth outcomes (Goisis et al. 2018; Myrskylä et al. 2017). Likewise, no significant difference in physical and mental functioning and positive child-parent interactions (Kim et al. 2018) seems to exist between older mothers and their younger counterparts. “Older” mothers generally feel more “ready” and less stressed by having children and this results in higher levels of happiness. Hence, mothers of advanced age do not seem to have reduced parenting capacity (Steiner and Paulson 2007). On the contrary, children born to older mothers often have higher cognitive and non-cognitive abilities and better socio-economic positions in their adult lives (Barclay & Myrskylä 2016; Carslake et al. 2017; Myrskylä et al. 2017). Finally, thanks to improved healthcare and lifestyles, life expectancy has increased steadily, with the result that is unlikely that “older” mothers, aged 40 or older, would die before their children reach the age of maturity.

A third approach used to oppose the objections against advanced motherhood it to rely on a series of classical philosophical arguments. The first, very famous one in the context of reproductive decision-making is the non-identity problem (NIP) of Derek Parfit (1984). It concerns the moral question of how our actions influence the life and identity of future, unborn children. Postponing pregnancy is an example of an identity-altering intervention insofar a child conceived when a mother is 40 will be a different child from the one that the same mother would have conceived at the age of 30. The NIP is sometimes used to show that *even if* late childbearing might involve increased health-related risks for offspring, children born to older women have not been harmed by their mother and hence their decision to delay childbearing is not morally wrong (Goold & Savulescu 2009). This reasoning draws on Feinberg’s counterfactual comparative account (1984) of harm which states that a person is harmed if that person is made *worse off* than they would have been if the putatively harmful conduct would not have taken place. The child born to a 40-year-old mother is not “worse off” and thus cannot be harmed simply because they would not have existed if their mother would have got pregnant earlier. In fact, another child would have been born in its stead.

The second argument resembles the argument of marginal cases used in philosophy to defend the moral status of animals by comparing their mental capacities to those of “marginal cases” of humanity (e.g. infants, persons in a coma, people suffering from dementia) (Regan 1979; Singer 1995). This line of reasoning goes as follows: if rationality is the defining characteristic for denying moral status to animals, then we should deny that status also to such marginal cases because there is no morally relevant ability that those marginal-case humans possess, but animals lack. Therefore, if we are not justified denying moral status to those people, then animals must have moral status too. In the context of delayed childbearing a similar argument is used with regard to concerns about older mothers’ reduced parental capacity and longevity. Vulnerability, in fact, is not only a characteristic of “older” mothers but of younger ones (e.g. cancer survivors) too (Cutas & Smajdor 2015). Hence, those who reject advanced motherhood because of physical limitations or reduced life expectancy, should deny ART also to all those with a history of chronic illness or a disability. Since many people would consider the latter to be a blunt measure of discrimination, reduced physical ability and life expectancy should not play a role in the case of older mothers either (Cutas & Smajdor 2015).

A last important line of reasoning is an argument by analogy whereby perceived similarities—in this case between older parents and grandparents—are used to infer further similarities (Cutas & Smajdor 2015). Grandparents, and in particular grandmothers, are indispensable in many families: they are the major providers of informal child care for preschool children, especially when both parents are working and particularly in countries where child care is expensive and public child-care services and parental leave are lacking (Zamarro 2020). In some cases, grandparents become the child’s caregiver on a full-time basis because their parents cannot care for them (e.g. due to poverty; mental health concerns, domestic violence, etc.). The phenomenon of custodial grandparenting is becoming more widespread (Choi 2016) and is preferred over other types of placement such as non-kin foster care due to the perceived positive impact of certain factors on children’s wellbeing, e.g. contact with relatives and ties to cultural heritage (Hayslip 2005). So, if we generally accept that grandparents can effectively raise their grandchildren, despite their age, then why should we question the caregiving capacities of older parents?

However, each of these arguments can be criticized as they contain some important lacunae. There is, for example, profound disagreement about the ethical significance of the NIP (Doolabh et al. 2019). A commonly held view among philosophers is that just because a choice is identity-altering, the choice thereby does not become less morally problematic; what matters is the actual overall wellbeing. In other words, mothers have the moral obligation to make decisions that give their (future) children the best possible future, *independently* of whether they are made better or worse off compared to children that would have been born when the mother was younger. Moreover, this view seems to match public’s moral intuitions. Research, in fact, shows that the NIP does not seem to play a major role in the general public’s moral decision-making (Doolabh et al. 2019).

The argument from marginal cases can be criticized as well (Salomon 2010). In this line of reasoning, “older” women and younger women with chronic health conditions are flattened into one group by emphasizing what they lack, namely relevant traits for good motherhood. The unhappy consequence is that the right to access to ART of one group is pit against that of the other (Taylor 2017). It can be questioned whether this “philosophical exploitation” of young(er) women with for example a chronic illness is really needed to make a case for advanced motherhood or whether it would not make more sense to argue against the idea that young, abled mothers are more valuable than others (Taylor 2017).

Finally, the grandparent-analogy argument does not fully hold up either. While grandparents, and particularly grandmothers, are more involved in informal childcare compared to the past, unlike parents they tend to invest time in activities aimed at entertainment and enjoyment rather that in those that give the child a competitive advantage in the future (Harman et al. 2022). Also, grandmothers tend to disengage from the role of educators since they consider this to be a parental responsibility: they try to be present without interfering in the way their children raise their grandchildren (Harman et al. 2022). Grandparents who take on kinship care for their grandchildren are generally considered to be the “second best” option. These grandparents often feel pressured into a role with important negative personal impacts (Hingley-Jones et al. 2020). Hence, grandparents do raise their grandchildren, but their contributions are role-specific and context dependent.

## A new way of thinking about the ethics of advanced motherhood

### A way out of the impasse?

The positions of supporters and opponents have increasingly become entrenched, causing a deadlock in the discussion surrounding the ethics of advanced motherhood. Opponents can be accused of using children’s best interest as a smoke screen to detract attention away from their biological essentialism and ageist, ableist and sexist positions that continue to regulate women’s bodies and lives. Supporters of advanced motherhood, on the other hand, can be criticized for placing too much emphasis on these socio-cultural power structures (i.e. ableism, sexism, ageism) that dominate the female body and therefore to overlook the painful reality of fertility decline for many women. It is certainly true that before the mid-twentieth century childbearing at “older” age was a common and accepted social practice, which was only subsequently transformed into a medical condition posing health risks for both mothers and children (Baldwin 2019, Friese 2008). The narrative of the biological clock, in fact, emerged in the late 1970s and early 1980s to regulate the female life course in the context of women’s participation in the work force (Díaz 2021). Recognizing and at the same time criticizing the pathologization of advanced motherhood is crucial because it enables us to dismantle stereotypes about women, mothers and the female body (Baldwin 2019; Cardin 2020). Still, despite its merits, such a focus on regulatory discourses is guilty of a certain disregard for the materiality of the body (Bühler 2021); a disregard that resonates with the social model of disability for which society, and not physical impairment, is the main cause of exclusion for people with disabilities (Thomas 2007). But like a disabled person’s daily lived experience *is* shaped by their bodily impairment; women cannot avoid the fact that their reproductive potential *does* drop drastically in their late thirties. The reality of age-related fertility decline is not (merely) the result of discourses and social norms (Bühler 2021).

Where does this leave us? In order to move the debate forward, we need to find new ways of thinking about the ethics of advanced motherhood. For this purpose, we aim to revisit (reproductive) ethics through the lens of the figure of the so-called “older” mother. This means that rather than considering advanced motherhood as a “special” case, we aim to recast the standard reproductive debate by taking on the subject position of the “older” mum. This forces us to ask what is lacking in a debate that too often leads to the impasse described above.

Our critical approach is inspired by feminist philosophers Adriana Cavarero (2009, 2016) and Judith Butler (2004, 2016), and in particular by their reflections on the concept of vulnerability. For many feminist thinkers, discussions of corporeal vulnerability feel risky from the outset. For centuries, in fact, the unique biological processes of the female body have been the starting point and justification for women’s secondary status in society. Feminists had to fight hard to counter stereotypes of weakness and to show that much of women’s presumed vulnerability is socially constructed. However, the theme of bodily vulnerability enables us to raise questions on the relation between body, experience and language. In fact, for both Butler and Cavarero our corporeal vulnerability is more than just a physical vulnerability. For Cavarero it has to do with our *unique* embodiment; with the concrete materiality of *who* we are. In her insistence upon the primacy of the *who* (the uniqueness of every being) rather than on the *what* (the collective identity), Cavarero is influenced by phenomenology that foregrounds the lived experience of the body. Butler on the other hand contends that our vulnerability has something to do with a linguistic vulnerability and is thus clearly working from within a post-structuralist perspective that foregrounds the power structures that discipline the body. Considering the complexity of their work, we cannot engage with this scholarship in depth. However, we aim to combine the insights of both phenomenology and post-structuralism in order to engage in a process of ethical inquiry that questions some of the main assumptions in the debate on advanced motherhood. Concretely, this means two things. First, we want to bring “older” mothers to the forefront of the discussion. For this purpose, we want to think about these mothers as concrete, embodied and unique persons and thus ask ourselves *who* they actually are. Secondly, we want to bring the body back in the debate on delayed childbearing by exploring the interaction between biology and the socio-cultural context without reducing it to mere discourse.

### Moving away from the distorted image of older motherhood

So, let’s start with our first question: *Who* are the mothers that are we talking about within the context of advanced motherhood? To answer this question, we need to (a) correct the distorted and polarizing narratives about older mothers (who do/should we think about, when we think about older mothers?) and (b) listen more accurately to their unique voices (who are older mothers *in terms of their beliefs, experiences*?). We will address these two issues in turn.

In the EU, the share of birth to mothers over 40 has risen consistently over the last 20 years (Eurostat 2022). Today on average 1 out of 20 mothers is older than 40. In the medical context, such mothers are considered “old” as advanced age is usually defined as age 35 or more for the mother at the time of delivery of her baby (Cardin 2020). Yet, in public and academic discourse on advanced motherhood, critics often report on sensational stories of so-called grandmother moms; women who get pregnant through ART when they are in their 60 s or even 70 s (Caplan et al. 2010, Léchot & Glâveanu 2013, Skynews). This creates a discrepancy between actual older mothers and how common narratives depict them considering that grandmother moms—women in their 60 and 70 s—are, despite media sensation, a rather exceptional phenomenon.

Framing older mothers as ‘grandmother moms’—and portraying them as ridiculous, irresponsible, repulsive, or monstrous women (Adrian et al. 2021, Lahad et al. 2016, Scala & Orsini 2022)—generates intuitive reactions of disgust towards advanced motherhood, thus feeding an idea of wrongness. According to the bioethicist Leon Kass, (1997), reactions of disgust are elicited by a violation of the natural. The bias towards what is deemed “artificial” and thus abnormal (Shaw & Giles 2009), and the appraisal of what is perceived as natural and thus “normal” (Zwart 1994) may explain the undercurrent of moral repugnance towards advanced motherhood (Adrian et al. 2021). “Radiant” mothers in their forties and fifties often do not elicit the same repugnance as grandmother moms because they are able to pass for younger and thus to *appear* to be the child’s natural, biological mother (Bühler 2015). This may explain why opponents of advanced motherhood in their writings deliberately distort the image of older mothers by making reference to women in 60s and 70s. They take recourse to these extreme examples to elicit feelings of disgust and persuade others that they are right. But the question is: on what grounds does naturalness (and genetics) deserve special moral protection? (Cutas & Smajdor 2015) It is generally accepted within (bio)ethics that we cannot deduce a normative conclusion (ought) from a natural fact (is). Hence, those that argue that advanced motherhood (though ART) is immoral because unnatural; are committing a logical fallacy. Discussing the ethics of advanced motherhood in relation to the question of naturalness thus only leads to polarization between two extremes (Smajdor et al. 2018).

This brings us back to the second part of our question: *Who* are these “older” mothers in terms of their lived experiences? Currently, there are very few studies that explore the lived experiences of “older” mothers or pregnant women or birthing persons (Aldrighi et al. 2016 and 2018, Baldwin 2019; Friese et al. 2008; George-Carey et al. 2021), and the great majority of these does not focus explicitly on 40+ mothers (but rather take 35 as a cutoff). Existing research suggests that those experiences do not match with many of the assumptions made in the literature on advanced motherhood. First, the inability to conceive and the difficulty of finding a suitable partner who is as equally committed to parenthood are for many 40+ women the main reason to “delay” motherhood rather than higher education and employment factors (Baldwin 2019). Secondly, these older mothers overwhelmingly report high levels of well-being, which stands in stark contrast with the dominant risk-centered approach to advanced motherhood (Steiner 2007; George-Carey 2021). Women who become mothers later onwards feel more prepared for motherhood, and perceive of themselves as being more responsible and patient (Aldrighi et al. 2016). Still, older mothers also often recount experiences of fear for their own and their child’s physical and mental health both during pregnancy and after birth (Aldrighi et al. 2016, 2018); feelings that seem to be triggered by the fact that healthcare professionals label their pregnancy as a high risk because of their age (Aldrighi et al. 2016 and 2018, Baldwin 2019). Older mothers also express ambivalent feelings regarding motherhood: emotions of pride, joy and gratitude go hand in hand with feelings of embarrassment and stigmatization caused by the perceived social prejudice surrounding late pregnancy (Aldrighi et al. 2016). Furthermore, healthcare professionals often tend to take on a paternalistic and authoritarian attitude towards older mothers because of their age (Aldrighi et al. 2016), i.e. they decide upon the kind of information to share and on the way of relaying it in order to ensure the choice of what they consider to be the best choice (Huschke et al. 2022). The often almost exclusive focus on the delivery of a healthy baby risks to disregard women’s experiences and feelings. Research shows that limited autonomy and ineffective communication often lie at the core of negative pregnancy and birth experiences which in turn might result in adverse psychological outcomes in mothers and thus cause lower quality of life postpartum (Huschke et al. 2022). Furthermore, the “special” attention reserved for older mothers highlights the idea that late pregnancy is somehow considered “abnormal”, and this might increase women’s anxiety (Aldrighi et al. 2016).

By bringing these experiences to the center of the discourse about advanced motherhood, it will be easier to break the tie between the divisive views of older mothers as either active and irresponsible risk seekers that ignore the dangers of late pregnancy, or passive individuals subject to unavoidable socio-cultural power structures. Understanding older mothers in their complexity and acknowledging this both in public discourse and in the healthcare context can be the key to deal with this multilayered phenomenon in an open manner, and make a shift from a medicalized, risk-based model to more person-centered care which enhances women’s involvement in decision-making.

Our research group is currently involved in an international and interdisciplinary research project on family building at advanced parental age (APA) in two contexts, spontaneous conception and medically assisted reproduction. One of the aims is to capture the experiences and attitudes of stakeholders (would-be parents of 40+, parents of 40+, their children and healthcare professionals) regarding responsible parenthood in relation to APA. In this way, our project inscribes itself in a more person-centered approach to advanced motherhood.

### Bringing the body back into the debate

As outlined in the introduction, we aim to revisit reproductive ethics through the lens of the “older” mother. This means that motherhood at advanced age is not considered a “special” case or set apart as something “abnormal”, but rather that older mothers are placed at the center of our ethical thinking. For this reason, we aim not only to listen to their needs and experiences, but also to pay attention to their bodies.

To do this, one must consider that life expectancy in industrialized countries has increased dramatically over the last decades: from about 50 years at the beginning of the twentieth century to almost 80 years nowadays. This explains why cultural ideas about ageing and the ideal life course have changed: discrete and age-defined life course transitions—such as the transition to parenthood—are being dismantled in favor of more flexible views (Friese et al. 2016). In fact, in many western countries the average age of women at birth is increasing as more and more women opt to delay motherhood until later in life. Still, not all women are able to become mothers at an advanced age. It is an undeniable biological fact that women’s reproductive potential is limited: their fertility potential drops when they are in their mid-late thirties and menopause commonly sets on between the age of 45 and 55 (Duncan et al. 2018). Thus whereas thanks to medical progress life expectancy has increased considerably, the length of women’s reproductive life span has not changed much as treatments to slow or reverse reproductive ageing are lacking. It can thus easily be established that women pass more of their life being infertile than fertile (Garrison 2021).

The desynchronization of reproductive and somatic aging explains why mothers over the age of 35 are considered “geriatric” by the medical community: They are considered “old” not because of their chronological age as such, but because of ovarian ageing (i.e. reduction in oocyte quantity and quality). Human ovaries, in fact, age more quickly—even 5 times faster—than any other female organ or tissue, including the uterus (Amanvermez et al. 2016; Yureneva et al. 2021). Although male fertility is somehow impacted by age, men do not have a fertile window as sperm is continuously formed. Iin principle, men are thus able to reproduce throughout their entire lifespan. Studies show that women (and men) often underestimate the impact that age has on their ability to conceive, both naturally and by means of assisted reproduction (Dougall et al. 2013; Delbaere et al. 2020, Fauser et al. 2019). Fertility education programs aim to increase people’s awareness and to allow them to make informed reproductive choices (Harper et al. 2021). Yet, research shows that even with more information, many women are unable to anticipate motherhood due to personal-life circumstances (Dougall et al. 2013). Hence, we are confronted with the following question: Why do we accept that, as a result of declining ovarian function, the time window for women to have children closes around the same time that they find a suitable partner, obtain financial stability and develop their careers (Not Aging 2022)? Moreover, the onset of menopause does not only affect fertility, but also leads to poor overall health, e.g. increased risk for heart disease, stroke, arthritis, depression, cognitive decline and so on (Ji et al. 2015, Zhu et al. 2019). Why then has the topic of ovarian ageing for a long time been overlooked (Garrison 2021; Nat Aging 2022)? Might it be due to the persistent gender bias in medicine and medical research?

There’s a long history of the female body being chronically understudied due to women’s underrepresentation as subjects in clinical research with serious, negative effects on diagnoses and treatment (Hallam et al. 2022; Plevkova et al. 2020). The outdated idea that male and female bodies can be treated the same, except when it comes to reproductive health, was framed “bikini-medicine” by the American cardiologist Nanette Wenger. Still, female reproductive and sexual health have only seemingly received more attention than other aspects of women’s health (Hayssen 2020). Endometriosis, for example, a chronic gynecological disease, affecting 1 in 10 women that can cause severe pelvic pain and infertility, continues to be vastly understudied and underfunded (Zale et al. 2020). Likewise, important gaps of care exist for female sexual dysfunction (SD) (e.g. loss of desire, decreased arousal, pain with sexual activity), a condition which often results in personal distress and reduced quality of life for those women who are affected by it (Nappi et al. 2016). Currently there are only two drugs on the market to treat female SD which got approved in 2015 and 2019. Both treatments are modestly effective and have important potential side effects (Shapiro et al. 2017; Simon et al. 2019).

Currently research on ovarian ageing and its effect on both fertility and general health however is gaining momentum (Nat Aging 2022). For example, in 2018 the Buck Institute for Research on Aging created a new center for Female Reproductive Longevity and Equality. The program aims to allow women to continue childbearing until their mid-50 s and at the same time reduce the morbidity and mortality risks associated with menopause and ovarian ageing (Gulbranson and Garrison 2020). Other promising initiatives both in academia and the industry are being undertaken (Nat Aging 2022). Still, more research is needed as the exact mechanisms responsible for ovarian aging are still incompletely understood (Yureneva et al. 2021; Das and Destouni 2023; Nat Aging 2022).

In light of the many structural and socio-cultural factors that influence women’s reproductive decisions and given the negative impact of the onset of menopause on women’s overall health, allocating resources in research to better understand ovarian ageing is in our view not only ethically permissible, but might even be ethically desirable if we want to establish health-related equality, or even just to reconcile with the increasing difference between somatic and reproductive ageing. That does not mean that preventive actions such as awareness and education campaigns to counter both the underestimation of age-related fertility decline and the overestimation of ART success rates are no longer necessary, it rather means that reducing the number of late pregnancies by advising women to have children earlier in life cannot be the only goal.

## Conclusion: challenges and possible objections

Our call for a better understanding of ovarian ageing might be vulnerable to the following objections: (1) it contributes to the continuing medicalization of women’s bodies; (2) it can be classified as a form of human enhancement as it violates “normal” biological processes; and (3) it risks to reinforce the social reproductive imperative that defines womanhood through (genetic) motherhood and thus promotes a pronatalist and geneticist stance.

Medicalization is often defined as the process by which some aspects of human life that were not considered pathological, are turned into medical problems (Conrad 2007). Medicalization is generally considered to have widespread negative effects. Treatment for menopause is sometimes considered to be a prime example of medicalization (Howard 2021): from being a biological process that every woman will experience in her adult life, it is turned into a deficiency disease. Increasingly calls are made for resisting this unnecessary medicalization as it reinforces the stigma and shame attached to menopause and ageing. We, too, are concerned about middle-ageism and the commercial exploitation of menopause. On the other hand, we think it is naïve to understand de-medicalization as the mere opposite of medicalization and to attribute a self-evident binary normative meaning to them (i.e. medicalization is bad and de-medicalization is good) (Horstkötter et al. 2015). De-medicalization, for example, might cause healthcare providers to trivialize symptoms (as is manifest in the word choice to describe menopausal symptoms: hot flashes and mood swings) and to underestimate the very real suffering women experience during this life phase, leaving them feeling ashamed and isolated (Jamieson 2020). Promoting a better understanding of the underlying factors of ovarian ageing to prevent and treat adverse long-term outcomes might lead to less medicalization later on and thus be beneficial for women’s overall health. Moreover, research on ovarian ageing to expand the reproductive lifespan will lead to less invasive fertility treatments and thus be beneficial to women’s physical and mental health.

Research on ovarian ageing might be used to prolong women’s reproductive potentiality until later in life, and thus to postpone the natural onset of menopause. As such, it challenges to a certain extent the body’s natural biological limitations and might be considered a form of human enhancement: it aims to improve human functioning even if there is no real pathology to be treated and thus to subvert human nature. Still, recent evolutionary studies seem to suggest that menopause is not an invariable natural trait, but is still evolving and can even disappear completely due to shifting patterns of mate choice and marriage at later age (Chan et al. 2020). Moreover, contrary to ART treatments that *circumvent* rather than actually *treat* the etiologic factors of infertility (Bell 2019), research on female reproductive longevity actually aims to tackle the problem of ovarian ageing by “treating” or “curing” dysfunctional ovaries. As such, it is interested more in a restorative practice aimed at the return to health rather than in enhancement aimed at improvement. Moreover, this type of research might also offer valuable insights to counter depletion of ovarian reserve in young women. Hence, rather than pitting the rights of one group of women against the other, it gives rise to what the political scholar Claire Jean Kim (2015) has called an ethics of mutual avowal; i.e. the recognition of unity between positions without privileging the one over the other. Moreover, given the significant morbidity associated with the onset of menopause, studies on female reproductive longevity can be used to sustain women’s health over time. Hence, considering all the above factors together, it is difficult to classify research on female reproductive ageing as being invested in “true” enhancement.

Finally, investing in research on ovarian ageing to mitigate the impact of female reproductive ageing on women’s reproductive choices and to give women the possibility to have children later in life, does not mean to say that biological motherhood is superior to other forms of motherhood or that motherhood should be a desirable goal for all women/persons. In fact, given that this type of research also aims to reduce morbidities associated with menopause, it will be of value to *all* women independently of whether they want to have children (later) in life or not. Secondly, although we urgently need more counter-narratives on family-making to combat geneticism (Gotlib 2016), for some women this research might represent the *only* option to become mothers at later age given that in some countries oocyte donation is not allowed, oocytes are scarce, and adoption is a very time-consuming, expensive and ethically charged process (Cutas & Smajdor 2015). Moreover, if research on ovarian ageing becomes more mainstream it might contribute to the further de-stigmatization of late pregnancies, very much like the availability and accessibility of ART seem to have positively influenced social attitudes about childbearing at later age (Lee et al. 2019). Finally, if older women will be able to use their own oocytes, they do not longer need to rely on donor eggs, avoiding the risk of exploiting socially and economically marginalized women.

## Data Availability

Not applicable.
